# Endogenous Interleukin-17a Contributes to Normal Spatial Memory Retention but Does Not Affect Early Behavioral or Neuropathological Outcomes after Experimental Traumatic Brain Injury

**DOI:** 10.1089/neur.2022.0017

**Published:** 2022-09-01

**Authors:** Dennis W. Simon, Itay Raphael, Kendall M. Johnson, C. Edward Dixon, Vincent Vagni, Keri Janesko-Feldman, Patrick M. Kochanek, Hülya Bayir, Robert S.B. Clark, Mandy J. McGeachy

**Affiliations:** ^1^ Department of Critical Care Medicine, University of Pittsburgh School of Medicine, Pittsburgh, Pennsylvania, USA; ^2^ Department of Pediatrics, University of Pittsburgh School of Medicine, Pittsburgh, Pennsylvania, USA; ^3^ Department of Medicine, University of Pittsburgh School of Medicine, Pittsburgh, Pennsylvania, USA; ^4^ Department of Neurological Surgery, University of Pittsburgh School of Medicine, Pittsburgh, Pennsylvania, USA; ^5^ Department of Environmental and Occupational Health, University of Pittsburgh School of Medicine, Pittsburgh, Pennsylvania, USA; ^6^ Department of Clinical and Translational Science Institute, University of Pittsburgh School of Medicine, Pittsburgh, Pennsylvania, USA; ^7^ Department of Safar Center for Resuscitation Research, University of Pittsburgh School of Medicine, Pittsburgh, Pennsylvania, USA; ^8^ Children's Neuroscience Institute, Children's Hospital of Pittsburgh, Pittsburgh, Pennsylvania, USA

**Keywords:** head trauma, interleukin, lymphocyte, neuroinflammation

## Abstract

Interleukin-17 (IL-17) is a proinflammatory cytokine primarily secreted in the brain by inflammatory T lymphocytes and glial cells. IL-17^+^ T-helper (Th17) cells are increased in the ipsilateral hemisphere after experimental traumatic brain injury (TBI), and IL-17 levels are increased in serum and brain tissue. We hypothesized that *il17a* and related gene expression would be increased in brain tissue after TBI in mice and *il17a^–/–^* mice would demonstrate neuroprotection versus wild type. The controlled cortical impact (CCI) model of TBI in adult male C57BL6/J mice was used for all experiments. Data were analyzed by analysis of variance (ANOVA) or repeated-measures two-way ANOVA with the Bonferroni correction. A value of *p* < 0.05 determined significance. Expression of *il17a* was significantly reduced in the ipsilateral cortex and hippocampus by day 3 after TBI, and expression remained low at 28 days. There were no differences between *il17a^–/–^ and il17a^+/+^* mice in beam balance, Morris water maze performance, or lesion volume after CCI. Surprisingly, naïve *il17a*^–/–^ mice performed significantly (*p* = 0.02) worse than naïve *il17a^+/+^* mice on the probe trial. In conclusion, sustained depression of *il17a* gene expression was observed in brains after TBI in adult mice. Genetic knockout of IL-17 was not neuroprotective after TBI. IL-17a may be important for memory retention in naïve mice.

## Introduction

The Centers for Disease Control and Prevention estimates that 1.7 million persons suffer from traumatic brain injury (TBI) in the United States each year and 5.3 million persons have TBI-related disability.^1^ In survivors, TBI is linked to chronic neuroinflammation, progressive neurological deficits, and neurodegenerative disorders.^2–4^ Two-year follow-up after moderate-severe TBI in children revealed a significant reduction in quality of life, participation in activities, and ability to communicate and care for themselves.^5^ An improved understanding of the immune mediators that impact outcome from TBI may allow for the development of targeted immunomodulatory therapies to attenuate secondary neurological injury and improve recovery.^2^

Interleukin-17 (IL-17) is a proinflammatory cytokine with receptors on neurons and glial cells in the central nervous system (CNS). The primary function of IL-17 is to bind to non-hematopoietic cells in target tissues and induce an innate-like immune response, including the induction of chemokines and cytokines that act to protect the host from microbial invasion.^6^ Humans with genetic defects in IL-17 signaling have increased the susceptibility to infection. Chronic activation of IL-17-mediated inflammation contributes to pathological autoimmunity, which has been well described in psoriasis and various forms of arthritis. In the CNS, major sources of IL-17 include T-helper 17 (Th17) cells and γδ-T cells and minor sources include astrocytes, microglia, and tissue-resident macrophages.^7,8^

Translational studies have established a key role of IL-17 and IL-17-producing T lymphocytes in the pathophysiology of multiple sclerosis (MS), stroke, and traumatic spinal cord injury through effects on blood–brain barrier permeability, cellular infiltration into inflamed or damaged tissues, and immune cell activation.^7,9–12^ The anti-IL-17 antibody, secukinumab, has undergone successful phase II trials in patients with MS.^6^ Recent publications have identified a key role for IL-17A in beta-amyloid (Aβ)-related neurodegeneration; however, this has not been investigated in a TBI model that accelerates Aβ deposition.^3,13–15^

Despite the body of literature on IL-17 impacting outcome in other neurological diseases, there have been only limited investigations on the role of IL-17 on outcome from TBI.^16^ In a murine model of TBI, Th17 polarization driven by M1-like macrophages peaked in the brain and blood at 24 h after injury and persisted significantly above sham levels out to 3 weeks from injury.^12,16^ In a study of pharmacological inhibition of the IL-17 axis in a TBI model, Li and colleagues reported reduced neuronal apoptosis and attenuated neurological deficits.^17^

Using the controlled cortical impact (CCI) model of TBI in adult mice, we performed a time course of *il17a* and related gene transcripts up to 28 days from injury and assessed motor function and spatial memory acquisition in wild-type (WT) and *il17a*-knockout naïve versus injured mice. Here, we report depressed IL17a gene expression between 3 and 28 days after injury, no significant change in motor or cognitive outcomes between WT and *il17a*-knockout mice after TBI, and a significant reduction in *il17a* gene expression in the ipsilateral cortex and hippocampus up to 28 days after injury. Surprisingly, we report that naïve *il17a*^–/–^ mice exhibit impaired spatial memory retention versus naïve *il17a^+/+^* mice, suggesting a possible role for *il17a* in normal memory retention.

## Methods

### Animals and surgical procedures

All studies were approved by the Institutional Animal Care and Use Committee of the University of Pittsburgh (Pittsburgh, PA). WT and age-matched *il17a*^–/–^ C57BL6 mice were purchased from The Jackson Laboratory (Bar Harbor, ME) and allowed to acclimate for at least 1 week before surgical procedures. Mice were housed in a temperature-controlled room with lighting on a 12-h day/night schedule with access to standard chow and water *ad libitum*. The CCI model of TBI was performed as previously described.^18^

Briefly, 12- to 16-week-old male mice (25–30 g) were anesthetized with 4% isoflurane in a 2:1 mixture of nitrous oxide and oxygen and then maintained at 1–2% after induction. Mice were placed into a custom stereotactic frame with ear bars to stabilize the head. The scalp was shaved and prepped with 10% povidone-iodine solution. A temperature probe was inserted into the temporalis muscle ipsilateral to the injury and temperature maintained at 37°C ± 0.5°C. The skin was then incised and a 5-mm craniotomy performed over the left parietal cortex. A pneumatic impactor device with a 3-mm flat tip was used to produce the CCI with two injury levels: A 5-m/s velocity, 1.2-mm depth, 50-msec dwell was used for reverse-transcriptase polymerase chain reaction (RT-PCR) studies to produce a moderate injury to preserve hippocampal tissue for analysis, and a 6-m/s, 1.8-mm depth, 50-msec dwell was used to produce a behavioral deficit on the Morris water maze (MWM). After CCI, the bone flap was replaced and secured with Koldmount dental cement (Vernon-Benshoff, Albany, NY), the skin was sutured closed, bupivacaine 0.25% was applied to the wound topically, and mice were placed in a chamber and provided supplemental oxygen for 30 min before the return to standard housing. Because of the inflammatory response that results from a craniotomy in mice,^16^ naïve mice had no surgical manipulation performed.

Mice were assessed daily for their well-being by Safar Center for Resuscitation Research and Department of Laboratory Animal Research staff.

### RNA isolation and quantitative polymerase chain reaction

At specified time points after CCI, mice were anesthetized with 4% isoflurane and perfused by cardiac puncture with 30 mL of cold heparinized saline. The brain was then carefully removed and the ipsilateral cortex and hippocampus were dissected and snap frozen for RNA extraction. RNA was isolated with RNeasy Mini kits (QIAGEN, Frederick, MD), following the manufacturer's protocol, and reverse-transcribed to complementary DNA with Superscript III First Strand kits (ThermoFisherScientific, Waltham, MA), according to the manufacturer's protocol. *Il17a*, RAR-related orphan receptor C (*rorc*), and interferon-gamma (*ifng*) genes were measured by real-time RT-PCR (qPCR) using SYBR Green Master mix (2 × ) with ROX (Invitrogen, Carlsbad, CA) on a 7300 Real Time PCR (Applied Biosystems, Waltham, MA) and RT^2^ qPCR Primers (QIAGEN). Expression fold-change was determined using the delta-delta cycle threshold method and normalized to beta-actin. The following primers were used: NM_010552 RT^2^ qPCR Primer for mouse il17a; NM_011281 RT^2^ qPCR Primer for mouse Rorc; NM_008337 RT^2^ qPCR Primer for mouse Ifng; and NM_007393 RT^2^ qPCR Primer for mouse Actb.

### Functional outcomes

Motor function was assessed using a beam balance as previously described.^19,20^ Briefly, mice (*n* = 10 per group) underwent training on day 0 to remain on a 1.5-cm-wide and 90-cm-high round wooden beam for 60 sec for three consecutive trials. Mice were then tested on days 1–5 by an observer blinded to the experimental group, and the average time spent on the beam over three trials on each day was recorded.

Spatial memory acquisition was assessed using the MWM as previously described.^20,21^ Briefly, mice (*n* = 10 per group) underwent testing on days 14–18 after injury for latency to find the hidden platform. Probe and visible platform trials were performed on days 19–20. The pool was located in a 2.5 × 2.5 m room with visual cues and lighting that were kept consistent throughout the experiment.

### Histological assessment

At 21 days after injury, after completion of behavioral testing, mice were reanesthetized and perfused with 30 mL of cold heparinized saline followed by 30-mL of 10% paraformaldehyde. The brain was cryoprotected in 15% and 30% sucrose solution and snap frozen in liquid nitrogen. Serial coronal 10-μm sections were taken every 500 μm through the brain using a cryotome and stained with hematoxylin and eosin. Three coronal sections were taken at each interval, and the average lesion volume for the three sections was used. Image analysis software (MCID; Imaging Research, Saint Catherines, Ontario, Canada) was used to analyze lesion volume, as previously described, by an evaluator blinded to the treatment group.^22^ Additional 10-μm sections were taken through the dorsal hippocampus. One section through the dorsal hippocampus per animal was used for each primary antibody. Immunohistochemistry was performed using a standard technique as previously described.^21^

Briefly, sections were blocked with 3% normal goat serum (Vector Laboratories, Burlingame, CA) in Tris-buffered saline containing 0.25% Triton X for 1 h. Sections were incubated overnight with the following primary antibodies: anti-Abeta40 (#44-136; ThermoFisherScientific); anti-Abeta42 (#44-344; ThermoFisherScientific); anti-synatophysin (ab14692; Abcam, Cambridge, MA); anti-Iba1 (ionized calcium-binding adaptor molecule 1; Wako Chemicals, Osaka, Japan); and anti-CD8a (clone 4SM16; ThermoFisherScientific). Sections were then incubated for 1 h with antirabbit horseradish peroxidase–conjugated secondary antibody (ABC Elite Kit; Vector Laboratories) and stained with 3,3’-diaminobenzidine (Vector Laboratories). A Nikon Eclipse 90i microscope (Nikon Corporation, Tokyo, Japan) was used for imaging.

### Statistical analysis

Data were analyzed using Prism software (version 8; Graphpad Software, La Jolla, CA). Gene expression was analyzed by analysis of variance (ANOVA) with Bonferroni's correction for multiple comparisons. Behavioral data were analyzed by paired *t*-test, Mann-Whitney U test, or repeated-measures two-way ANOVA as appropriate. When the overall ANOVA revealed a significant effect, the data were further analyzed with the Bonferroni correction for multiple comparisons to determine significant group differences. Data are presented as mean ± standard error of the mean or scatter plots of individual data points ± standard deviation as appropriate. A *p* value <0.05 was considered significant.

## Results

### Interleukin-17a gene expression is reduced in the ipsilateral cortex and hippocampus after traumatic brain injury

**FIG. 1.**
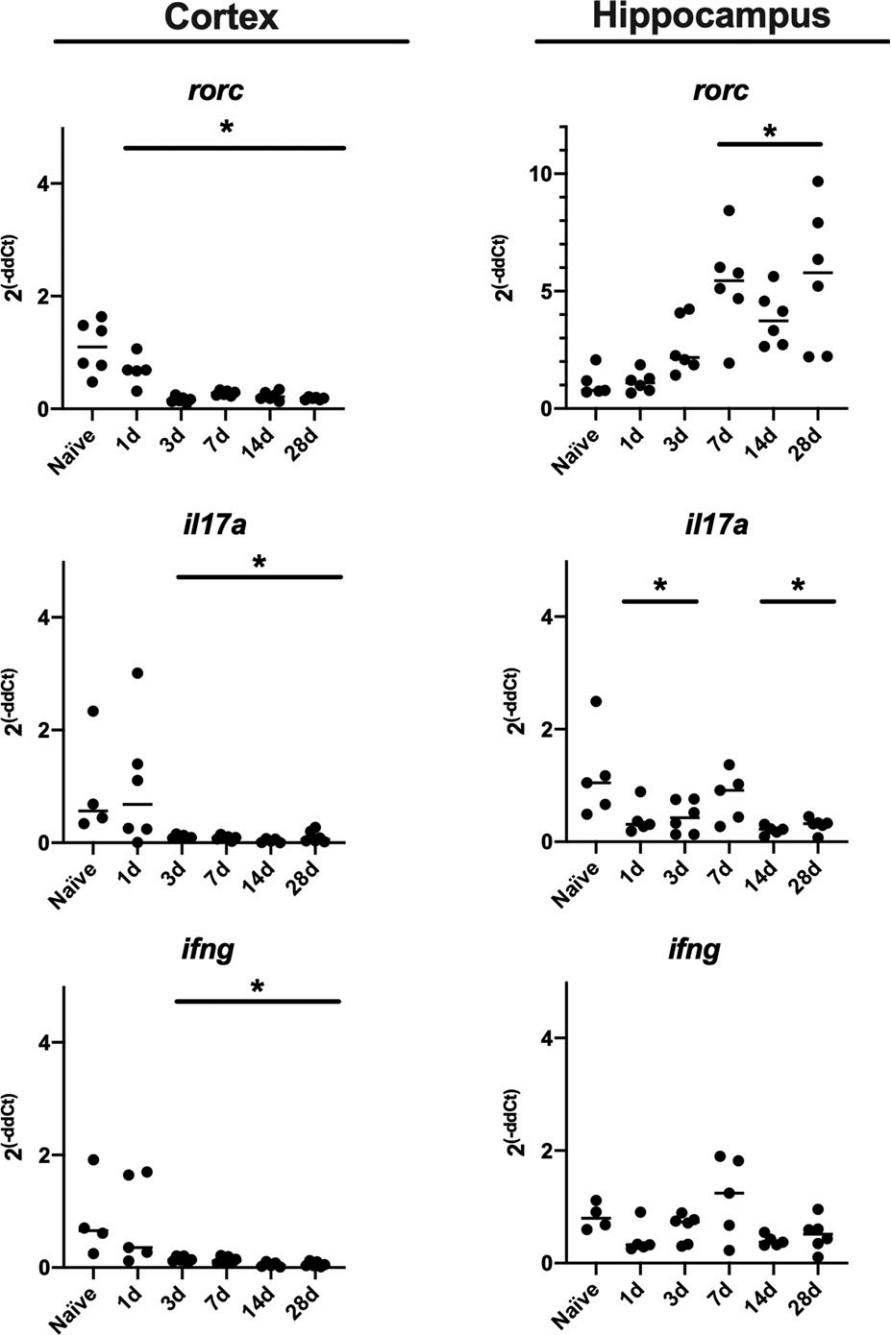
Expression of *il17a* and related genes after TBI in mice. At specified time points after TBI, mice (*n* = 4–6 per time point) were euthanized and the ipsilateral cortex and hippocampus isolated for RT-PCR analysis for *il17a* and related genes. Shown is relative expression relative to beta-actin. Data were analyzed by ANOVA with a Bonferroni adjustment for multiple comparisons. *p < 0.05; **p < 0.001. ANOVA, analysis of variance; *il17a*, interleukin-17a; RT-PCR, reverse-transcriptase polymerase chain reaction; TBI, traumatic brain injury.

We performed qPCR to determine the relative expression of *il17a* and related genes after TBI (*n* = 4–6 mice per time point). The *rorc* gene was selected because it encodes RAR-related orphan nuclear receptor gamma t, the master transcription factor expressed by Th17 cells and IL-17-producing innate cells. The *ifng* gene encodes the cytokine, IFNγ, the signature effector molecule of Th1 cells that is also produced by proinflammatory Th17 cells. In the cortex, there was a reduction in expression of *rorc* starting at 1 day after injury and continuing out to 28 days after injury when compared to naïve mice. Consistent with this finding, expression of *il17a* and *infg* were reduced starting 3 days after TBI (Fig. 1). In the hippocampus, expression of *rorc* was increased between 7 and 28 days after injury. Surprisingly, expression of *il17a* did not correspond to *rorc* and was reduced on days 1, 3, 14, and 28 after TBI, and no change was observed in *ifng* expression between injured and naïve mice.

### No difference between il17^–/–^ and wild-type mice in beam balance test after traumatic brain injury

**FIG. 2.**
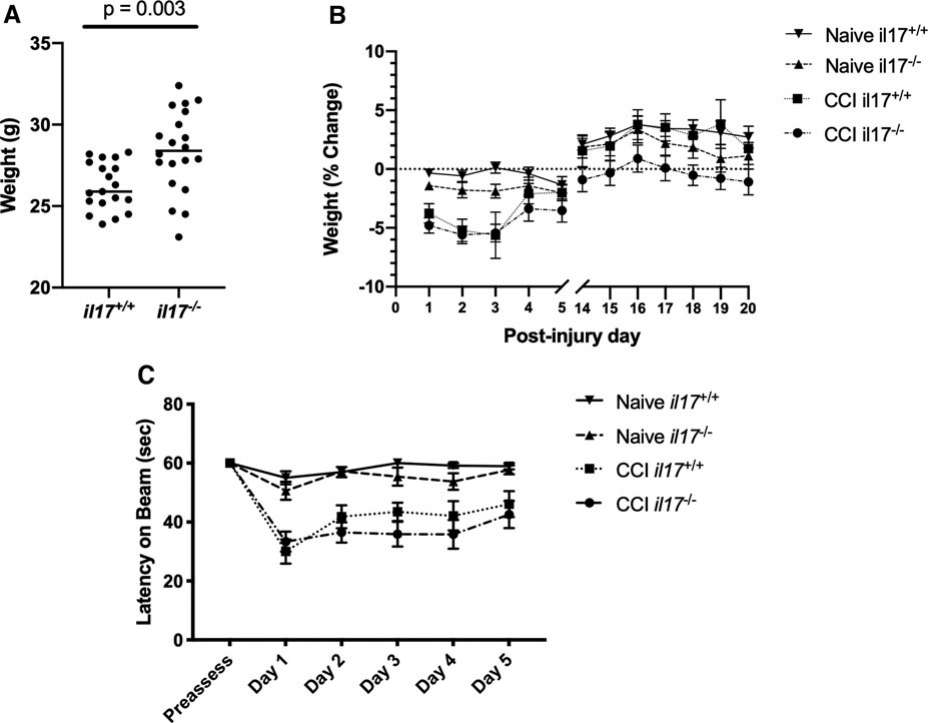
Motor function testing in wild-type and IL-17-knockout mice after TBI. (**A,B**) Wild-type and *il17*^–/–^ mice (*n* = 10 per group) had weight measured before CCI and then daily during motor and cognitive testing. (**C**) Mice were trained to remain on a circular beam balance for 60 sec before CCI. After CCI, mice were tested on days 1–5 for latency to remain on the platform. Each mouse had the average of three trials taken for each day. Data were analyzed by paired *t*-test or repeated-measures two-way ANOVA with a Bonferroni adjustment for multiple comparisons as appropriate. ANOVA, analysis of variance; CCI, controlled cortical impact; IL-17, interleukin-17; TBI, traumatic brain injury.

To assess fine motor and coordination, mice were randomized and trained to maintain balance for 60 sec on the beam for three trials before TBI. Compared to age-matched WT mice, *il17^–/–^* mice had increased body mass, as expected^23^ (Fig. 2A). After randomization and TBI, WT and *il17^–/–^* mice lost and regained weight at similar rates (*n* = 10 per group; Fig. 2B). There was a significant injury effect of TBI on ability to remain on the beam (*p* < 0.001). However, there was no difference between naïve *il17^+/+^* and *il17^–/–^* mice or injured *il17^+/+^* and *il17^–/–^* mice (Fig. 2C).

### No difference between il17^–/–^ and wild-type mice after traumatic brain injury in Morris water maze

**FIG. 3.**
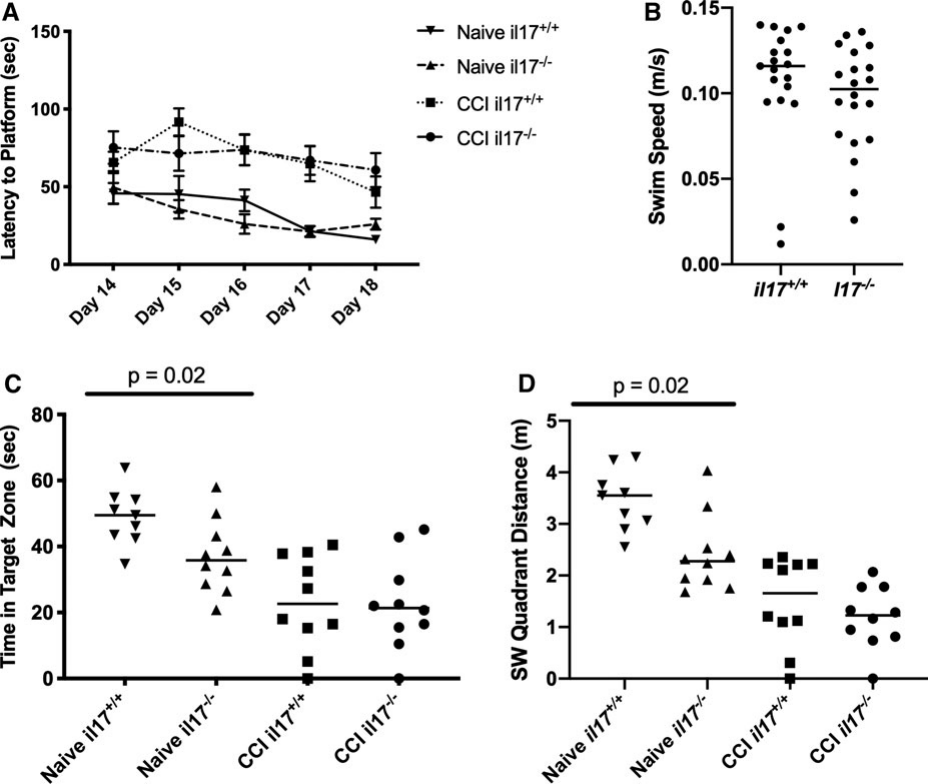
Spatial memory acquisition in wild-type and IL-17-knockout mice after TBI. Wild-type and *il17*^–/–^ mice (*n* = 10 per group) were studied using the Morris water maze on days 14–20 after CCI. (**A**) Latency to find a submerged platform using visual cues was measured on days 14–18. (**B**) Latency to find a visible platform was measured on days 19–20. (**C,D**) On day 20, the platform was removed and time and distance traveled in the target quadrant was measured. Submerged platform performance was analyzed by repeated-measures two-way ANOVA with Bonferroni's correction for multiple comparisons. Visible and probe trial performance was analyzed by one-way ANOVA with Bonferroni's adjustment for multiple comparisons. ANOVA, analysis of variance; CCI, controlled cortical impact; IL-17, interleukin-17; TBI, traumatic brain injury.

To assess hippocampal spatial memory acquisition, we used the MWM. On days 14–18 after TBI mice (*n* = 9–10 per group) were assessed for their ability to find a hidden platform using visual cues. An injury effect was observed between naïve and injured *il17^+/+^* mice (*p* < 0.001). However, no significant differences were observed between *il17^+/+^* and *il17^–/–^* mice (Fig. 3A). To confirm that there were no effects attributable to genotype on swim speed, we compared mean swim speed between groups (Fig. 3B) and observed no difference. For the probe trial, we compared time in the target zone as well as distance traveled in the target zone. In both cases, there was no difference between *il17^+/+^* and *il17^–/–^* mice after TBI (Fig. 3C,D).

Interestingly, naïve *il17^–/–^* mice performed significantly worse than WT controls by both metrics (*p* = 0.02), suggesting that IL-17A plays a role in the generation of long-term spatial memory. To determine whether IL-17 was related to the integrity of synapses, levels of the synaptic marker, synaptophysin, were examined in the ventral hypothalamus (Supplementary Fig. S1).^15^ No significant difference was observed with CCI or between WT and *il17^–/–^* mice. IL-17 has been shown to impact the development of dementia-related pathologies.^13^ We performed immunostaining for Aβ on mice euthanized on day 21 after CCI (Supplementary Fig. S2). We did not observe Aβ plaques in WT or *il17^–/–^* groups.

### No difference in lesion volume between il17^–/–^ and wild-type mice after traumatic brain injury

**FIG. 4.**
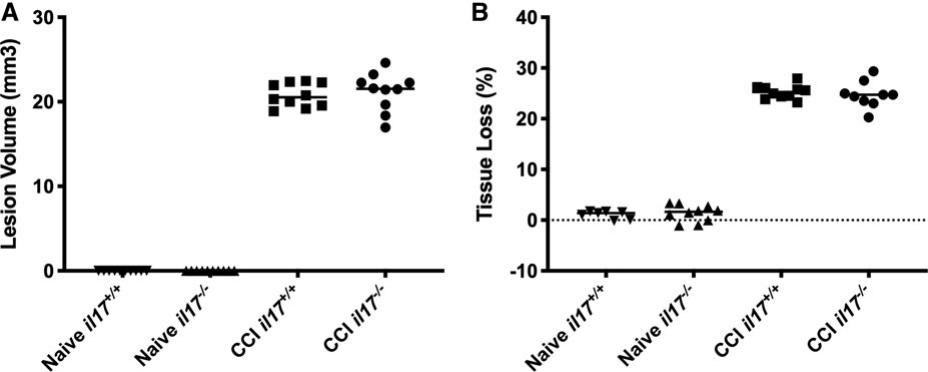
**(A)** Lesion volume and **(B)** percent tissue loss in wild-type and IL-17-knockout mice after TBI. Mice (*n* = 10 per group) were euthanized on day 21 after injury, and lesion volume and percent tissue volume loss were assessed by an investigator blinded to group assignment. Data were analyzed by ANOVA with a Bonferroni adjustment for multiple comparisons. ANOVA, analysis of variance; CCI, controlled cortical impact; IL-17, interleukin-17; TBI, traumatic brain injury.

After completion of MWM, on day 21 mice (*n* = 9–10 per group) were euthanized and lesion volume was assessed. After TBI, no significant differences were observed between *il17^+/+^* and *il17^–/–^* mice in either lesion volume or percentage of tissue volume loss (Fig. 4A). Notably, there was also no significant difference in total hemispheric tissue loss between naïve *il17^+/+^* and *il17^–/–^* mice (Fig. 4B), corresponding to similar injury-induced deficits in performance.

### Compensatory increase in infiltrating CD8^+^ cells in il17^-/-^ mice after TBI

**FIG. 5.**
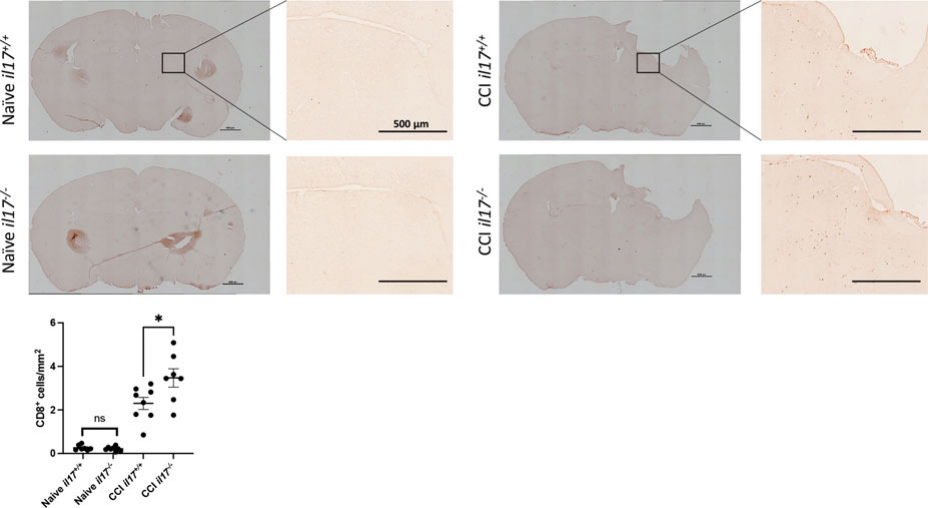
Increased CD8^+^ cell infiltration after TBI in IL-17-knockout mice. Wild-type and *il17*^–/–^ mice (*n* = 8–10 per group) were euthanized on day 21 after injury, and 10-μm coronal sections 1.82 mm from bregma were stained for CD8a. CD8^+^ cells were counted in both hemispheres, and the data were analyzed by one-way ANOVA with a Bonferroni adjustment for multiple comparisons. *p < 0.05. Inset scale bar = 500 μm. ANOVA, analysis of variance; CCI, controlled cortical impact; IL-17, interleukin-17; TBI, traumatic brain injury.

To assess for compensatory increase in CD8^+^ cells in *il17^–/–^* mice, we assessed CD8^+^ cell infiltration histologically. No difference was observed between naïve WT or *il17^–/–^* groups. After CCI, *il17^–/–^* had a greater number of CD8^+^ cells present (Fig. 5). IL-17 has been shown to promote microglial activation. We performed immunostaining for Iba-1 on mice euthanized at day 21 after CCI (Supplementary Fig. S3). There was a significant increase in Iba-1 staining after CCI; however, no difference was observed between WT and *il17^–/–^* mice.

## Discussion

Local and systemic inflammatory response to TBI are complex,^2,24^ and thus far the translation of immune-directed therapies to clinical practice has been disappointing.^2,25^ Genetic or pharmacological blockade of IL-17 has been reported to reduce lesion volume and enhance recovery in models of acute ischemic stroke.^9,26^ However, IL-17 can also promote wound healing in the skin,^27^ bone,^28^ and gut.^29^ Reduced *il17a* expression suggested some level of regulation of the IL-17 response after TBI. It was somewhat surprising to find that IL-17 deficiency had no clear effect on either lesion size or cognitive deficits in the CCI model of TBI.

Studies of the IL-17 axis in the brain after TBI^12,16,17,30–32^ have primarily focused on relative changes in T-cell polarization or IL-17 protein levels in brain tissue homogenates performed as part of a larger cytokine multiplex. Thus, little has been previously known about *il17a* gene expression in the brain after TBI. Although we found *il17a* expression reduced in the ipsilateral cortex and hippocampus after TBI, the impact on IL-17 protein levels or IL-17 activity over time was not determined. In our study we addressed acute time points with an investigation of *il17a* gene expression between 1 and 28 days after TBI and an assessment of motor function, spatial memory acquisition, and lesion volume up to 21 days after injury. Although our approach mirrored the overwhelming majority of pre-clinical studies in TBI, a more delayed assessment of outcome is also warranted.^12,16^ It is also possible that, at these time points, our gene expression analysis did not detect a relative increase in IL-17-producing cells attributable to our removal of meningeal tissue rich in lymphocyte populations before brain homogenization, or the relative paucity of IL-17-producing lymphocytes in brain tissue after TBI compared to other neuroinflammatory conditions.^6^

We observed an increased expression of *rorc* in the hippocampus between days 7 and 28 after injury. The *rorc* encoded transcription factor, ROR-gamma, promotes the differentiation of Th17 cells. Recently, Daglas and colleagues hypothesized that Th17-like cells in the hippocampus may promote CD8^+^ cytolytic T-cell-mediated damage at chronic time points.^16^ Daglas and colleagues, using a CCI model, demonstrated that granzyme B^+^CD8^+^ accumulation in the brain contributes to chronic motor impairment. An earlier increase of IL-17- and IFN-γ-producing CD4^+^ T cells may have contributed to the cytotoxicity of CD8^+^ cells. In our study, *il17^–/–^* mice had a compensatory increase in CD8^+^ cells after CCI relative to *il17^+/+^* mice without a difference in acute motor function or spatial memory acquisition. Possibly, genetic knockout of *il17* reduced the cytotoxic activation of infiltrating CD8^+^ T cells and attenuated the chronic neurological impairment that Daglas and colleagues observed.

Neutralizing approaches to block IL-17 post-TBI may be more effective for neuroprotection than a constitutive IL-17 knockout. Investigating the role of IL-17 in TBI, Li and colleagues^17^ used a daily injection of the histone deacetylase inhibitor, suberoylanilide hydroxamic acid (SAHA), which had been previously shown to block Th17 differentiation and IL-17 production, and investigated apoptotic cell death and modified neurological severity score (mNSS) out to 7 days from injury. They observed significant protection in mice treated with SAHA versus vehicle at 3 and 7 days after injury. Although we did not observe a similar result indicating neuroprotection on beam balance assessment over this time period, the beam balance represents only one part of the mNSS performed by Li and colleagues. Also in contrast to Li and colleagues, who assessed apoptotic cell death in the first week after injury, we performed an assessment of lesion volume. Future studies could investigate the role of IL-17 in neuronal cell death using the CCI contusion model of TBI. It is also a consideration that the neuroprotective effects of SAHA were not attributable to IL-17 inhibition given that it would be expected to have multiple effects on immune cells and gene expression in general.

Alternatively, a low-level expression of IL-17 caused by an inhibitor such as SAHA may be preferable to genetic deletion of the *il17a* gene. For example, studies of the roles of inducible nitric oxide synthase (iNOS) and tumor necrosis factor (TNF) after TBI have demonstrated how proinflammatory mediators may have different effects on outcomes linked to neuroprotection depending on the time points chosen. Both iNOS and TNF had been associated with acute cellular damage and neurological deficits after TBI. However, longer-term studies subsequently demonstrated late neuroprotection in mice deficient of iNOS and TNF signaling.^22,33^ Similarly, increased levels of IL-1β are associated with secondary brain injury after TBI, but low levels are important for recovery processes such as neurogenesis.^2^ Therefore, it is possible that a more complete neurological assessment performed within the first week, or a more delayed neurological assessment performed after 5 weeks, would demonstrate a difference between WT and *il17^–/–^* mice after TBI.

Additionally, the study by Li and colleagues used the weight-drop model of TBI, and, though it is difficult to make comparisons across models, injury level was likely less severe than the injury level in our CCI experiments to obtain a deficit on the MWM. The type of injury model as well as the severity of injury impact the immune response,^2,25^ perhaps resulting in the opposing IL-17 findings between the two studies.

Trauma-related neurodegenerative disease, including Alzheimer's disease, is estimated to account for 3–10% of dementia.^34^ There is currently no effective treatment for Alzheimer's disease. The role of IL-17 in the pathogenesis of Alzheimer's disease has been assessed using the triple-transgenic Alzheimer's disease and intracerebroventricular (i.c.v.) injection of Aβ_1-42_ models.^14,35^ IL-17^+^ cells were shown to accumulate in the brain at the onset of neurodegenerative symptoms. Neutralization of IL-17 with an i.c.v. injection of anti-IL-17 antibody attenuated neurodegeneration and synaptic dysfunction and improved memory function, possibly independent of Aβ pathology. We performed immunostaining for Aβ_1-40_ and Aβ_1-42_. At the time point we assessed, amyloid plaques were not observed in *il17^+/+^* or *il17^–/–^* mice. Further investigation of a role for IL-17 in early intracellular amyloid deposition after trauma is warranted.

In adult polytrauma patients, the IL-17 axis has been shown to be activated and associated with organ damage and mortality.^36,37^ In a trauma plus hemorrhagic shock model, administration of anti-IL-17a antibody resulted in a marked reduction in alanine aminotransferase.^36^ The isolated CCI model requires that efforts are made to avoid modifiable factors associated with secondary injury, including fever, hypotension, and hypoxemia. However, it is possible that the study of a systemic inflammatory mediator such as IL-17 may be more impactful using combined neurotrauma models,^38^ such as the combined TBI plus hemorrhagic shock model,^39^ and such an approach would also be relevant given the important role of secondary insults after severe TBI in humans.

Our preliminary finding of reduced time and distance traveled in the target quadrant on the probe trial in naïve *il17^–/–^* mice suggests a role for IL-17 in spatial memory retention. This is similar to the finding by Takemiya and colleagues^40^ on the role of IL-1β on the MWM and probe trial. Unlike IL-1β, *il17a* genes have not been reported to be expressed in association with long-term memory or hippocampal neurogenesis. However, novel roles for IL-17 outside of traditional inflammation and involving neurocognitive function have been proposed. For example, Choi and colleagues^41^ reported autistic-like behavior in pups born to mothers with elevated systemic IL-17, and Beurel and colleagues^42^ reported increased susceptibility to depression in mice after the administration of Th17 cells, which demonstrated trafficking to the hippocampus, but not bulk lymphocytes. Ribeiro and colleagues^43^ found that mice deficient for meningeal IL-17^+^ γδ T cells had impaired hippocampal synaptic plasticity and short-term memory in the Y-maze and MWM paradigms, but did not observe any differences using the standard MWM protocol for long-term memory. Although γδ T cells have been investigated in ischemic brain injury, little is known about their role in TBI. Synaptic plasticity and short-term memory could be rescued with exogenous administration of brain-derived neurotrophic factor. A role for IL-17 in long-term hippocampal memory formation under normal physiological conditions may be demonstrated by our probe trial data.

Although we did not detect a difference in swim speed or motor function between *il17a*^–/–^ and *il17a^+/+^* mice after TBI, we cannot rule out other contributors to probe trial performance such as motivation; however, we did not detect a difference in naïve mice between genotypes on the hidden platform component of water maze testing. Further studies to confirm these results and investigate mechanisms underlying the potential role of endogenous IL-17 in memory processing are warranted.

## Conclusion

Expression of *il17a* was reduced in the ipsilateral cortex and hippocampus between 3 and 28 days after experimental TBI in male adult mice. *I1-17a* gene deletion also did not confer neuroprotection either on behavior or histology in the initial 21 days after TBI. Surprisingly, naïve *il17a*^–/–^ mice exhibited impaired spatial memory retention versus naïve *il17a^+/+^* mice, suggesting a possible role for *il17a* in normal memory retention. Further investigations should focus on the role IL-17 may play on longer-term outcomes from TBI and exploration of its possible role in memory processing.

## Supplementary Material

Supplemental data

Supplemental data

Supplemental data

## Abbreviations Used

Aβ: amyloid-beta
ANOVA: analysis of variance
CCI: controlled cortical impact
CNS: central nervous system
Iba1: ionized calcium-binding adaptor molecule 1
i.c.v.: intracerebroventricular
ifng: interferon-gamma
IL-17: interleukin-17
iNOS: inducible nitric oxide synthase
mNSS: modified neurological severity score
MS: multiple sclerosis
MWM: Morris water maze
rorc: RAR-related orphan receptor C
RT-PCR: reverse-transcriptase polymerase chain reaction
SAHA: suberoylanilide hydroxamic acid
TBI: traumatic brain injury
Th17: T-helper 17
TNF: tumor necrosis factor
WT: wild type
