# Supplementary material for: Endogenous Interleukin-17a Contributes to Normal Spatial Memory Retention but Does Not Affect Early Behavioral or Neuropathological Outcomes after Experimental Traumatic Brain Injury
Source: Neurotrauma Rep. 2022 Sep 1;3(1):340–51. doi: 10.1089/neur.2022.0017 (PMC9531893; doi:10.1089/neur.2022.0017)

**Supplemental Figure 2. Dementia-related pathologies in wild type and IL-17 knockout mice after TBI.** Wild-type and *il17*^-/-^ mice (n=8-10/group) were sacrificed on day 21 after injury and 10μm coronal sections -1.82mm from bregma were stained for β-amyloid (anti-Abeta40, anti-Abeta42). No β-amyloid plaques were observed.


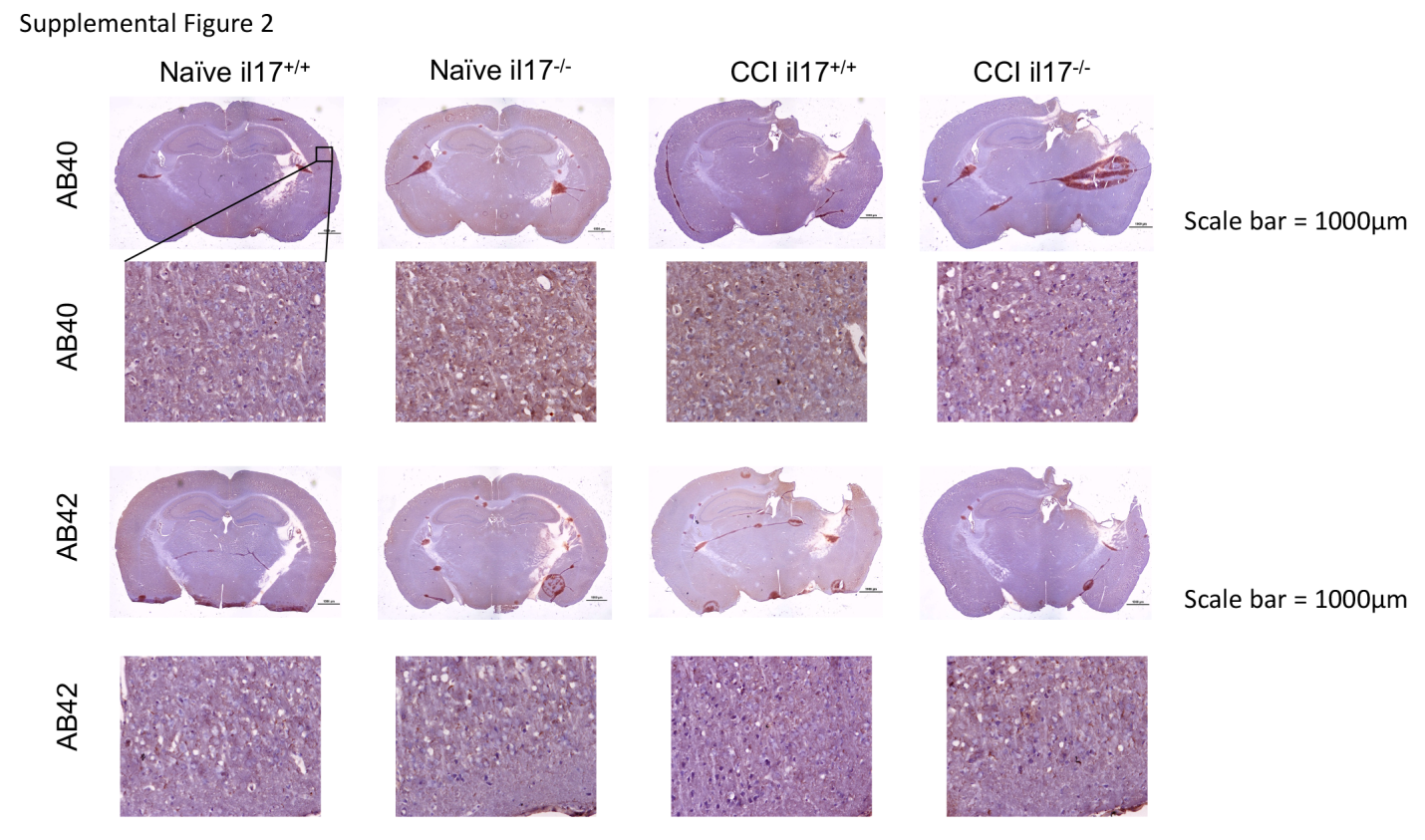

Supplement: Supplemental data [file Supp_FigS2.docx]
