# Supplementary material for: Endogenous Interleukin-17a Contributes to Normal Spatial Memory Retention but Does Not Affect Early Behavioral or Neuropathological Outcomes after Experimental Traumatic Brain Injury
Source: Neurotrauma Rep. 2022 Sep 1;3(1):340–51. doi: 10.1089/neur.2022.0017 (PMC9531893; doi:10.1089/neur.2022.0017)

**Supplemental Figure 3. Microglial activation in wild type and IL-17 knockout mice after TBI.** Wild-type and *il17*^-/-^ mice (n=8-10/group) were sacrificed on day 21 after injury and 10μm coronal sections -1.82mm from bregma were stained for ionized calcium binding adaptor molecule 1 (IBA-1). Percent area was calculated in the pericontusional cortex, thalamus, and ventral hypothalamus. Data analyzed by one-way ANOVA with Bonferroni adjustment for multiple comparisons.


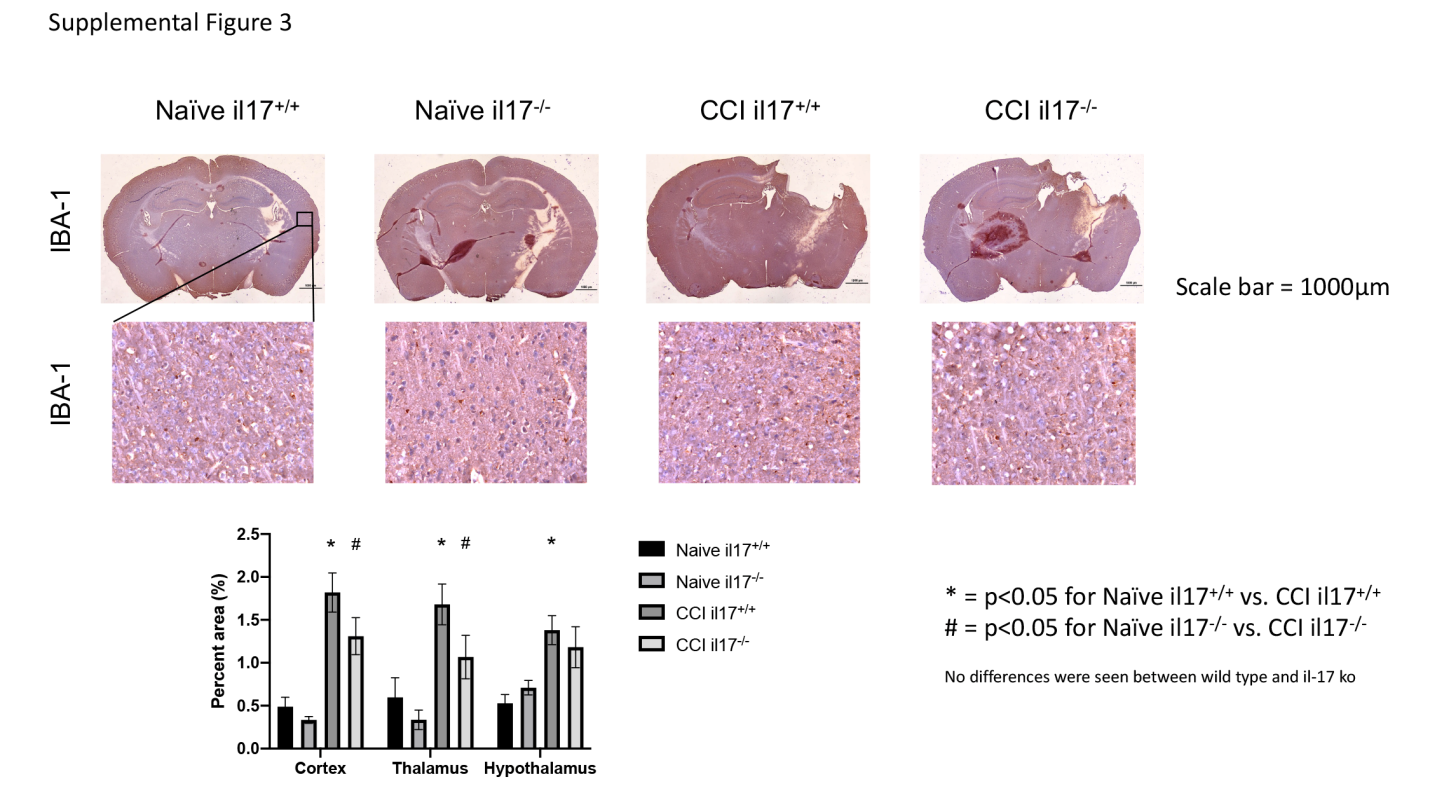

Supplement: Supplemental data [file Supp_FigS3.docx]
